# Effects of various mastitis treatments on the reproductive performance of cows

**DOI:** 10.1186/s12917-020-02305-7

**Published:** 2020-03-30

**Authors:** Sebastian Smulski, Marek Gehrke, Kacper Libera, Adam Cieslak, Haihao Huang, Amlan Kumar Patra, Malgorzata Szumacher-Strabel

**Affiliations:** 1 Department of Internal Diseases and Diagnosis, Faculty of Veterinary Medicine and Animal Science, Poznań University of Life Sciences, Poznań, Poland; 2 Veterinary Centre, Nicolaus Copernicus University, Torun, Poland; 3 Department of Preclinical Sciences and Infectious Diseases, Faculty of Veterinary Medicine and Animal Science, Poznań University of Life Sciences, Wolynska 35, 60-637 Poznan, Poland; 4 Department of Animal Nutrition, Faculty of Veterinary Medicine and Animal Science, Poznań University of Life Sciences, Poznań, Poland; 5 Department of Animal Nutrition, West Bengal University of Animal and Fishery Sciences, Kolkata, India

**Keywords:** Mastitis, Cows, Reproduction indices

## Abstract

**Background:**

The purpose of the study described here was to evaluate the effects of different supportive treatments - such as antioxidants, immunomodulators, and nonsteroidal anti-inflammatory drugs (NSAIDs) - in mastitic cows treated with intramammary antibiotics on the efficacy of mastitis therapy and fertility indices. Fertility indices, including time to first insemination, conception rate, time between calving and conception (open days), and number of services per conception (insemination index), were evaluated for 300 dairy cows. Sixty cows without apparent clinical signs of mastitis were assigned 100 days after calving to a Control group. Another 240 cows with clinical mastitis were systematically divided into four experimental groups (I–IV) of 60 cows each. All mastitic cows were treated with approved intramammary antibiotics in recommended doses. Cows in Group I were treated with intramammary antibiotics only. Cows in Groups II, III, and IV, received intramammary antibiotic therapy and a single injection with antioxidants, an immunomodulator (lysozyme dimer), or an NSAID (flunixin meglumine), respectively.

**Results:**

The lowest treatment efficacy of mastitic quarters and cows was noted in Group I (51.6 and 53.3%; *p* > 0.05). The best recovery rate was noted in Group II (63.3 and 66.7%; *p* > 0.05), followed by Group III (58.3 and 60.9%) and Group IV (58.3 and 58.0%; *p* > 0.05). The above data did not differ statistically (*p* > 0.05). The animals with mastitis (Groups I–IV) showed prolonged time to first insemination, more open days, higher insemination index, and lower conception rate than the control cows (*p* <  0.05). The conception rate of healthy cows and of successfully treated cows was insignificantly lower than that of cows required prolonged antibiotic therapy. Supportive treatments improved the mastitis recovery rate compared with intramammary antibiotics only. The efficacy of mastitis treatments affected the reproduction indices: in cows requiring prolonged treatment with antioxidants, a shorter time to first insemination was needed than in other groups (*p* <  0.05). Fewer days open were observed between the group with antioxidants and the control group (*p* <  0.05).

**Conclusions:**

Clinical mastitis negatively affects reproductive indices (days open, pregnancy rate after first AI, NSC) in dairy cows. Different types of supportive medicine, such as antioxidants (vitamin C and E, and β-carotene), lysozyme dimer, or NSAID can be useful in improving fertility in mastitis cows treated with antibiotic only. It has been proven that each supportive treatment improved antibiotics efficiency and the antibiotic combined with the antioxidants was the most effective treatment.

## Background

Researchers are concerned about reduced reproductive performance in dairy cattle simultaneously with decreased milk yield. The antagonism between fertility and overall milk production has been emphasized by many authors who have examined production and fertility indicators over the years in the same herd [1–3]. For example, in the 1970s, the conception rate after first artificial insemination (**AI**) was between 50 and 60%; this has now declined to 35–45% due to increased milk yield [4, 5]. Knowledge of cattle reproduction and herd management has been improved in recent decades [6, 7]. Decreased fertility may be caused by management failures, indigestion, and metabolic disorders, which result from poor quality dietary components or negative energy balance, as well as from genetic selection focused mainly on milk yield [8].

The negative effect of subclinical and clinical form of mastitis on fertility and ovary functionality of cows has been well documented [9, 10]. Reactive oxygen species **(ROS)** released during mastitis development negatively affect reproductive systems, being responsible for decreased progesterone production and induced apoptosis of corpus luteum **(CL)** cells, resulting in adverse effects on oocytes [11, 12]. Physiological luteolysis is also caused by CL epithelium cells and is linked to the ROS produced by these cells. It is supposed that the ROS take part in local processes responsible for luteolysis [13]. Proinflammatory cytokines are released during inflammation, which stimulates the production of ROS and activates phospholipase A2, leading to luteolysis [14].

Moore et al. [15] suggested that clinical mastitis (CM) exerts an indirect impact on fertility by modifying the interestrus period and shortening luteal phase. Cows diagnosed with CM in the period from calving to first breeding were characterized by prolonged days open and more inseminations required for conception than were healthy animals. Santos et al. [16] stated that the occurrence of CM in the period from the first insemination decreased the efficiency of this procedure and lowered the conception ratio (CR), as well as increasing culling and the percentage of embryo deaths and abortion. According to Schrick et al. [10], not only can CM lead to delayed first insemination, extended days open, and more AI needed for conception, but the subclinical form also has significant effects. A meta-analysis conducted by Fourichon et al. [17] with data collected over several decades concluded that retained placenta, ovarian cysts, and metabolic disorders affect cows’ fertility. Other researchers have also evaluated the effects of mastitis on fertility, but no information about any coexisting disorders was presented [10, 16]. These researchers suggested that either udder inflammation as isolated process or mastitis as process, combined with other disorders, could negatively affect fertility. The main research objectives of this study were thus to 1) study the association between the time of occurrence of CM (expressed in days in milk; DIM) and the reproductive performance of the cows under commercial farm conditions; 2) to assess the impact of different protocols of supportive mastitis therapy on reproductive performance, udder inflammation treatment efficiency, and total antioxidant status (TAS) of peripheral blood during mastitis.

## Results

A total of 238 cows were diagnosed with clinical mastitis, with 283 quarters affected. Two cows were remove from the experiment due to the presence of yeasts or *Prothoteca* spp. in milk.

The prevalence of the most significant pathogens isolated from milk (*Streptococcus* sp. and *Staphylococcus aureus*) was similar in all treatment groups (Table 1). Only the percentage of milk samples with *S. aureus* was lower in group IV (−antibiotic +NSAID), though this was not a statistically significant difference. However, no better effects of mastitis treatment were observed in this group.

**Table 1:** Bacteria isolated from infected quarters (no statistical differences were noted)

Treatment	*Str.* sp.	*S. aureus*	CNS	G-	Others^a^
	n ^1^(%)	n ^1^(%)	n ^1^(%)	n ^1^(%)	n ^1^(%)
Group I (antibiotic)	32 (43.2)	16 (21.6)	11 (14.9)	13 (17.6)	2 (2.7)
Group II (antibiotic + antioxidants)	29 (40.3)	17 (23.6)	15 (20.8)	9 (12.5)	2 (2.8)
Group III (antibiotic + lysozyme dimer)	30 (43.5)	15 (21.7)	12 (17.4)	11 (15.9)	1 (1.4)
Group IV (antibiotic + NSAID)	30 (44.1)	8 (11.8)	15 (22.1)	9 (13.2)	6 (8.8)
Total	121^2^ (42.7)	56^2^ (19.7)	53^2^ (18.7)	42^2^ (14.8)	11^2^ (3.8)

^a^no growth, mixed infection, or other Gram-positive bacteria

*CNS* Coagulase-negative staphylococci

*G-* Gram-negative bacteria

^1^Percentage of isolated bacteria to microorganisms isolated in group

^2^Percentage of all isolated microorganism

After the initial antimicrobial treatment, 60.1% of quarters (58.4% of cows) had recovered. Prolonged treatment was essential for 39.9% of quarters (41.6% of cows). The most effective initial therapy (with recovery of 66.7% of quarters) was observed in the group receiving both antibiotics and antioxidants (Table 2). Administration of the immunomodulator led to recovery in 60.9% of quarters (58.3% of cows) and the NSAID treatment resulted in recovery in 58% of quarters (58.3% of cows). The group treated with intramammary tubes alone saw the lowest recovery rate, at 53.3% of quarters (51.6% of cows). The differences between the groups were not statistically significant (*p* > 0.05; Table 1).

**Table 2:** Effects of supportive therapy on the effectiveness of mastitis treatment^1^

Treatment	FT		PRO	
	n Cows (Qr)	% Cows (Qr)	n Cows (Qr)	% Cows (Qr)
Group I (antibiotic)	31 (40)	51.6 (53.3)	28 (34)	47.4 (45.9)
Group II (antibiotic + antioxidants)	38 (48)	63. (66.7)	22 (24)	36.6 (33.3)
Group III (antibiotic + lysozyme)	35 (42)	58.3 (60.9)	25 (27)	41.6 (39.1)
Group IV (antibiotic + NSAID)	35 (40)	58.3 (58.0)	24 (28)	40.6 (41.1)
Total	139 (170)	58.4 (60.1)	99 (113)	41.6 (39.9)

*FT* First treatment, *PRO* Prolonged treatment, *NSAID* Nonsteroidal anti-inflammatory drugs, *Qr* Quarters

^1^No statistical differences were noted among the groups

### Effect of mastitis on fertility

Reproductive performance indices in the cows suffering from CM were significantly poorer than in the healthy cows (Table 3). Differences in the number of days open were also observed among those cured after the first treatment, those cured after prolonged treatment, and the control group (*p* <  0.05). Differences were observed between the member of Groups I (antibiotic only) and II (antibiotic with antioxidants) who required an additional treatment of antibiotics. Additionally, the time to first AI (days) was longer in the experimental groups than in the control group (*p* <  0.05). Cows requiring prolonged antioxidant treatment had a shorter time in days to first insemination than those in other groups requiring additional prolonged antibiotics treatment (*p* <  0.05). On the other hand, statistical differences for all treatment groups for the number of services per conception were not observed. In experimental antibiotic groups, there were 31 days open more compared to control group (*p* <  0.05). Those cows for whom first therapy or prolonged treatment was essential were characterized by more days open than the control group (average 24 days and 44.1 days more, respectively; *p* <  0.05). Cows cured after first or prolonged treatment showed more days to first AI and more days open than the control (*p* <  0.05; Table 3). Statistical differences were also observed between prolonged treatment with antibiotic + lysozyme dimer, prolonged treatment with antibiotic + NSAID, and the control group (41.7 and 43.5 respectively, *p* <  0.05). Those cows receiving additional treatment had fewer days to first AI and fewer days open, but the difference was not statistically significant.

**Table 3:** The effect of supportive therapy on time to first artificial insemination (AI), days open (DO) and service index per conception (S/C)

Group	n	AI			n	DO			n	S/C		
Control	60	78.5^*c*^	±	20.9^*m,z,c*^	60	88.8	±	27.4^*n*, z, *c*^	60	1.4	±	0.6^*m*, z, *c*^
Antibiotics	157	92.4	±	27.1^*y*^	157	119.8	±	43.7^y^	157	1.9	±	1.0 ^y^
First treatment	92	90.2	±	25.8^*k*^	102	112.8	±	38.2^*m*^	102	1.7	±	0.9 ^*m*^
Prolonged treatment	65	95.6	±	28.8^*k*^	54	132.9	±	50.3^*k*^	54	2.3	±	1.1^*k*^
First treatment												
Antibiotic	20	102.6	±	28.9 ^*ab*^	25	130.1	±	37.5^*ab*^	25	1.8	±	0.8^*abc*^
Antibiotic + antioxidants	25	78.6	±	21.9 ^*c*^	25	99.16	±	34.6^*bc*^	25	1.8	±	0.9^*abc*^
Antibiotic + lysozyme dimer	23	82.0	±	24.6 ^*bc*^	24	101.8	±	27.5^*bc*^	24	1.7	±	0.8^*bc*^
Antibiotic +NSAIDs	25	99.5	±	20.3 ^*abc*^	28	118.9	±	43.5^*abc*^	28	1.6	±	1.1^*bc*^
Prolonged treatment												
Antibiotic	21	108.9	±	31.4 ^*a*^	16	155.7	±	64.8^*a*^	16	2.4	±	1.3^*ab*^
Antibiotic + antioxidants	14	80.6	±	23.5 ^*bc*^	14	109.3	±	46.7^*bc*^	14	1.9	±	0.9^*abc*^
Antibiotic + lysozyme dimer	14	89.3	±	29.5 ^*abc*^	13	130.5	±	43.0^*ab*^	13	2.7	±	1.3^*a*^
Antibiotic +NSAIDs	15	97.1	±	23.1 ^*abc*^	12	132.3	±	26.0^*ab*^	12	2	±	0.6^*abc*^
SEM		1.78				2.86				0.06		
P value												
Control vs. first and prolonged treatments(^*k,m,n*^)		0.001				< 0.001				< 0.001		
Control vs. antibiotics (^y,z^)		< 0.001				< 0.001				< 0.001		
Control vs. treatments(^*a,b,c*^)		< 0.001				< 0.001				< 0.001		

*n* Number of observations, *SEM* Standard error of mean

Superscripts *k,m,n* in the same column indicate significantly different between control versus first treatment and prolonged treatment (*P* < 0.05)

Superscripts *y,z* in the same column indicate significantly different between control versus antibiotics (*P* < 0.05)

Superscripts *a,b,c* in the same column indicate significant different between control versus treatment groups (*P* < 0.05)

Considering the effectiveness of the therapy, a higher number of services per conception (by 0.9) was noted in cows receiving prolonged therapy than in the control group (*p* <  0.05). Moreover, the highest number of services per conception (NSC) was observed in the group with prolonged therapy combined with injection of the lysozyme dimer (2.7 ± 1.3). Lower NSC was observed in the group treated repeatedly with antibiotics alone (2.4 ± 1.3). Statistical differences were observed only in NSC compared with the control group (*p* < 0.05). The average value of NSC in the groups treated with single antimicrobial therapy alone, in comparison to the groups treated with antioxidants, was similar (1.8 ± 0.8 and 1.8 ± 0.9, respectively). In addition, NSC data from the antibiotic + lysozyme dimer and antibiotic + NSAID groups were slightly better (1.7 ± 0.8 and 1.6 ± 1.1, respectively).

A higher number of services per conception was observed in the experimental groups than in the control cows. However, the differences between those cured by the first treatment and those only recovering after prolonged treatment were not statistically significant.

On the other hand, the pregnancy rate after first AI and NSC depending on timing of mastitis did not differ (*p* > 0.05) (Table 4). Statistical differences were observed in terms of days open between cows with mastitis detected between 85 and 100 days after calving and mastitis detected earlier (0–21, 22–42, 43–63, and 64–84).

**Table 4:** Timing of mastitis and its effect on pregnancy rate after the first artificial insemination (AI), number of services per calving (NSC), and days open

Time of mastitis (days *postpartum*)	n	Pregnancy rate after first AI (%)	NSC	Days open (d)
0–21	89	70.0	1.7	110.7^*b*^
22–42	22	66.7	2.0	113.3^*b*^
43–63	22	63.0	1.8	120.7^*b*^
64–84	14	62.5	2.0	128.2^*ab*^
85–100	5	50.0	2.7	160.3^*a*^
Total/average mastitis cows	152	66.9	1.8	116.1
SEM		2.33	0.07	3.0
*P*-value		0.44	0.13	0.013

*n* Number of observations, *AI* Artificial insemination, *NSC* Number of services per calving

Different superscripts in the same column indicate significant difference (*p* < 0.05)

Only 20% of the cows who recovered from mastitis (the experimental group) became pregnant after the first AI within 70 days after parturition (average at 56.5 ± 6.3 day *postpartum*; Table 5). In the control group, there was a higher pregnancy rate after the first AI (43%; *p* < 0.05) on day 70 after parturition, with comparable AI timing (60.5 ± 6.7 days). The best results in the time to the first effective AI were observed in those treated with antioxidants. The proportion of cows that became pregnant within 70–90 days *postpartum* was comparable in both groups (33% in the group with mastitis and 30% in the control group). More cows become pregnant over 90 days after parturition in the experimental groups (50%) than in the control group (23%). The average number of days from calving to first AI was comparable in both groups of cows that became pregnant between 70 and 90 days *postpartum* (81.1 ± 6.1 days/group with mastitis; 81.4 ± 6.1 days/group of healthy cows) and later (113.2 ± 20.2 days/group with mastitis; 107.6 ± 16.4 days/group of healthy cows; *p* > 0.05). A tendency was observed for CR and NSC to be lower when mastitis was diagnosed close in time to breeding, though the data did not differ statistically.

**Table 5:** Number of cows with average number (and percent) of days to first artificial insemination in different groups

Group	AI (d)								
	< 70 d			70–90 d			> 90 d		
Control	60.5^a,j^	±	6.7	81.5	±	6.1	107.6	±	16.4
	26		43%	20		33%	14		23%
Experimental	56.5^k^	±	6.3	81.1	±	6.1	113.2	±	20.2
	31		20%	47		30%	79		50%
Antibiotic	61.3^ab^	±	5.1	82.7	±	5.9	121.0	±	24.9
	4.0		9%	10.0		23%	27		63%
Antibiotic + antioxidants	54.4^b^	±	6.0	82.3	±	5.6	103.5	±	11.5
	14		36%	12		31%	13		33%
Antibiotic + lysozyme	58.8^ab^	±	7.2	79.7	±	6.4	113.5	±	20.3
	12		32%	12		32%	13		35%
Antibiotic + NSAID	60.3^ab^	±	9.5	82.1	±	5.3	110.0	±	15.7
	3		8%	11		30%	26		70%
Total	58.4	±	6.7	81.2	±	6.0	112.4	±	19.7
	57		26%	67		31%	93		43%
SEM	0.89			0.74			2.05		
*P* value									
Control vs. experimental ^(j,k)^	0.026			0.84			0.33		
Control vs. groups ^(a,b)^	0.066			0.59			0.057		

We investigated the association between the timing of mastitis and the timing of AI and its efficiency. Table 6 show that all experimental cows were divided into eight groups based on the timing of mastitis in relation to the timing of first AI, and the calculated probability of conception for each group.

**Table 6:** Probability of conception after first AI, depending on timing of mastitis

^1^Group (n total = 213):	^2^Probability of conception:	*P*-value	n
Mastitis until 90 days before AI	0.97	0.92	169
Mastitis until 60 days before AI	0.78	0.39	100
Mastitis until 30 days before AI	0.63	0.25	34
Mastitis until 20 days before AI	0.67	0.43	20
Mastitis until 15 days before AI	0.66	0.44	17
Mastitis until 10 days before AI	0.60	0.44	11
Mastitis until 5 days before AI	0.39	0.21	10
Mastitis diagnosed either on day of AI or 15 days after AI	< 0.001	0.009	7

^1^Mean day of occurrence of mastitis before AI (30.0 ± 29.0); mean day of first AI (93.8 ± 27.9)

^2^Calculation based on logistic regression analysis

### The relation between mastitis and total antioxidant status

Table 7 shows the fluctuations in the total antioxidant status on days 0 and 7 from the onset of therapy, and the difference between the results on these 2 days.

**Table 7:** Total antioxidant status of blood (mM/L) on days 0 and 7 after mastitis diagnosis (mean ± SD)

Group	n	0			7			TAS changes		
Control	15	0.17	±	0.07	0.15	±	0.10^ab^	—0.02	±	0.09^j^
Cured	26	0.16	±	0.08	0.22	±	0.10	0.07	±	0.10^k^
Antibiotic +NSAID	8	0.14	±	0.06	0.15	±	0.06^ab^	0.01	±	0.11
Antibiotic + antioxidants	9	0.19	±	0.10	0.29	±	0.05^a^	0.10	±	0.08
Antibiotic + lysozyme	9	0.14	±	0.06	0.21	±	0.12^ab^	0.07	±	0.11
Prolonged	19	0.14	±	0.11	0.20	±	0.11	0.06	±	0.16^k^
Antibiotic +NSAID	7	0.12	±	0.07	0.12	±	0.11^b^	0.01	±	0.13
Antibiotic + antioxidants	6	0.15	±	0.19	0.28	±	0.08^a^	0.14	±	0.23
Antibiotic + lysozyme	7	0.14	±	0.06	0.19	±	0.09^a^	0.05	±	0.08
Total	60	0.15	±	0.09	0.19	±	0.11	0.04	±	0.12
SEM		0.011			0.014			0.016		
P value										
Control vs. cured and prolonged ^(j,k)^		0.55			0.12			0.07		
Control vs. treated groups ^(a;b)^		0.77			< 0.01			0.08		

*SEM* Standard error of means, *TAS* changes Total antioxidant changes from day 0 to 7

Different symbols for condition (control vs. cured and prolonged treatments [j,k]) and groups (control As. treated groups [a,b]) are indicated as significant differences (*P* < 0.05)

A slight increase in total antioxidant status (TAS) was observed in all groups on day 7 compared to day 0. The differences oscillated between 0.14 and 0.22 mM/L and did not differ significantly. There were significant differences in the dynamic of TAS changes from day 0 to 7 between the healthy and the prolonged groups. In conclusion, TAS fluctuations did not show any differences in data on day 0 and 7 of treatment, and would not reflect the relationship between the clinical assessment of the occurrence of mastitis and its therapeutic efficiency.

## Discussion

The effectiveness of mastitis therapy in this study fluctuated between 51.6 and 63.3% and was comparable to, or slightly lower than, the effectiveness of the therapy conducted by other authors. Milne et al. [18] reported that the efficiency of treatment of mastitis caused by streptococci was approximately 49%. In contrast, Malinowski et al. [19] noted that antibiotic therapy based on penicillin and neomycin had an effectiveness of 50%, and that amoxicillin with clavulanic acid and cefalexine with a single injection of lysozyme dimer had as much as 80% effectiveness against microorganisms such as *S. agalactiae, S. uberis, E. coli*, and *S. dysgalactiae*. In the present study, the lowest treatment efficiency was observed in the control mastitis group (51.6%), and the highest treatment efficiency was seen in the group treated with antioxidants (63.3%). The data did not differ significantly (*p* = 0.09). The literature has described the positive effect of antioxidants in mastitis therapy. Injections of vitamins C, E, and selenium 3 weeks before parturition increased total antioxidant status until 4 weeks after calving [20]. Moreover, supplementation with vitamin C decreased somatic cell counts in milk [21]. Beta-carotene is responsible for maintaining immunological functions, and can thus decrease incidences of mastitis and retained placenta [22]. Plasma beta-carotene concentration tended to be lowest in the first week after calving [23]. The reason for the better effectiveness of antibiotic therapy combined with vitamin E could be the positive correlation with increased chemotactic abilities in granulocytes. Oral and parenteral supplementation of this vitamin, when its serum plasma concentration reaches its nadir in the first 2 weeks after calving, can improve phagocytosis in blood granulocytes [24]. These results may indicate immunosuppression after calving, possibly due to decreased feed intake efficiency; this justifies the use of antioxidants in the period after calving.

The effectiveness of antibiotic treatment combined with lysozyme dimer was 58.3%, comparable with that of other methods of supportive therapy. The efficiency of this supportive treatment did not differ from its value in earlier experiments, where the antibiotic was used independently, and the effectiveness of therapy oscillated between 43.5 and 74.1%. In the study of Malinowski et al. [25], a single injection of lysosome dimer increased treatment effectiveness by 8.7%. In the present study, the use of lysozyme dimer increased the therapeutic efficiency by 6.7%, compared with the control mastitis group. However, the difference was not statistically significant (*p* = 0.36). The increased effectiveness of antibiotic therapy combined with lysozyme dimer in mastitis treatment has been observed in cows vaccinated against staphylococcus infections [26]. Malinowski et al. [25] noted a reduction in subclinical, bacterial, and nonbacterial udder inflammation cases after intravenous injection of lysozyme dimer.

In our experimental groups, the lowest efficacy of treatment was observed in the group of cows treated with the NSAID flunixin meglumine (58.3% of cows, 58% of quarters). The results of treatment were numerically higher (by 6.7% in cows and 4.7% in quarters) only than in the group of cows successfully treated with intramammary antibiotic only (*p* = 0.57). No improvement in antibiotic treatment combined with flunixin meglumine was described by Green et al. [27]. However, NSAID led to decreased body temperature with clinical signs of fever and reduced clinical signs of inflammation, especially in colimastitis [28]. Flunixin meglumine blocks the synthesis of PGF2α, which may have an effect on reproductive performance in cows with mastitis [29].

Fertility indicators did not differ significantly from the results obtained in other studies [16, 30]. The average number of days since parturition to first AI was 90.2 ± 25.8 days for cows cured after first treatment and 95.6 ± 28.8 days for cows needed prolonged therapy. The timing and variety of first AI date were similar to those obtained by Barker et al. [31]. In other studies [10, 16, 30], the time to first AI was shorter (66.0 to 77.3 days). Days open in the present study fell between 88.8 and 132.9 days, and was comparable with the result obtained by Schrick et al. [10] and Barker et al [31]. However, less promising results of 165 days open have been reported in another study [16]. In our study, the average number of services per conception was 1.4–2.3, which was similar to the results of other studies [10, 30].

The results suggest that the occurrence of mastitis 7–10 days before AI decreases insemination effectiveness. Most likely, prolonged treatment for more than 7–10 days and the on-going inflammation process (due to the low efficiency of microbial therapy or lack of supportive treatment) is the cause of this of negative influence of mastitis on fertility. Our results are coherent with those of Barker et al. [31] and Santos et al. [16]*,* who also noted a negative impact of mastitis either before or after AI on the number of days open. An increased culling rate among cows with mastitis close to AI, as well as the high proportion of embryo deaths, were also reported [16, 31]. Moreover, Ahmadzadeh et al. [30] noted longer open periods and higher insemination index in those cows in whom mastitis occurred between 56 and 105 days in milk and over 105 days in milk, as compared to cows with clinical signs of udder inflammation before 56 days *postpartum.* This study suggests that the occurrence of mastitis in the period close to AI has a clearer negative impact on fertility than does its occurrence in the early post calving period.

Considering the results of TAS in relation to treatment effectiveness, statistical differences were noted between the group with antioxidants who recovered after the first treatment in comparison to the group treated with prolonged therapy and NSAID. TAS values in all groups were comparable to those of other studies [32]. Analyzing TAS values does not seem to help assess either the intensity of inflammatory process or treatment efficiency. However, the administration of antioxidants in mastitis therapy results in increased effectiveness of the therapy, as has been confirmed in other studies through determination of TAS. This allows the negative impact of local inflammatory processes on fertility to be reduced [33]. Furthermore, when comparing these data, the conclusion could be drawn that the effectiveness of AI performed several days after the occurrence of mastitis may be reduced, and the high coefficient of determination confirms this. Thus, there is strong convergence between the curve and the calculated probability of conception.

## Conclusion

Considering the effect of the timing of mastitis after parturition on reproductive performance, the conclusion could be drawn that cows with udder inflammation during the first hundred days *postpartum* are characterized by longer time to first insemination, more days open, and a higher number of services per conception. Ineffective first antibiotic therapy can negatively affect the number of days open. The use of an additional single injection of antioxidants can improve the effectiveness of antibiotic treatment of mastitis, as well as the reproduction indices analyzed here.

## Methods

Three commercial dairy farms located in the middle of Poland were enrolled in this study. Management status of all enrolled farms was similar, being based on the free stall system. The average number of cows on each farm was approximately 300, with an average milk yield of about 7000 kg/lactation. A total of 240 dairy cows with the acute form of mastitis (hot and swollen udder, as well as macroscopic changes in milk, such as clots and watery discharge)—excluding cows with toxic mastitis (i.e., with systemic symptoms such as fever, recumbency, and retained fetal membranes)—were selected for this experiment. In the enrolled animals, CM was diagnosed from day 10 after parturition to day 14 after the first AI. Another 60 healthy cows were also allocated to this study. All these cows were divided into five groups, with four treatment groups and one control group (Table 8). On each farm, eighty mastitic cows (in four groups with twenty cows in each group) and twenty healthy cows were used. The treatment of mastitic cows in Groups I, II, III, and IV began immediately after the clinical diagnosis of mastitis. All mastitic cows were treated with approved intramammary antibiotic products. Intramammary tubes with proven antibiotic sensitivity (according to our laboratory tests) were used as recommended by the manufacturers. Cobactan (cefquinome sulfate 25 mg/1 ml; Intervet, Boxmeer, Netherlands) in farm A, Lincocin forte (lincomycin hydrochloride 330 mg/10 ml, neomycin sulfate 100 mg/10 ml; Pfizer, Animal Health, Belgium) in farm B, and Synulox (amoxicilin trihydrate 140 mg/ml, clavulanic acid 35 mg/ml, prednisolone 10 mg/ml Pfizer, Animal Health, UK) in farm C were used. All these intramammary antibiotic tubes were administrated three times every 12 hours. Additional supportive treatments were applied once, such as a single-shot intramuscular injection on the first day of antibiotic therapy. The following supportive preparations were used:

- antioxidants: vitamin C (Vitaminum C, Biowet Pulawy) at a dose of 0.01 mg/kg, β-carotene (Carofertin 10 mg/ml, Alverta u. Wertfft) at a dose of 0.5 mg/kg, vitamin E and Se (vitamin E 50 + Se Pro Inj) at doses of 1 mg/kg of vitamin E and 0.01 mg/kg of Se.
- immunomodulator: lysozyme dimer (5.0/10 ml Lydium-KLP, Nika Health Products) at a dose of 0.02 mg/kg
- NSAID: flunixin meglumine (50 mg/ml Flunimeg, Scanvet) at a dose of 2.2 mg/kg.

**Table 8:** Groups of cows and their treatment methods

Group	Cow condition (n)	Treatment method
I	Acute mastitis (60 cows)	Intramammary antibiotics only
II	Acute mastitis (60 cows)	Intramammary antibiotics + antioxidants (subcutaneous injection)
III	Acute mastitis (60 cows)	Intramammary antibiotics + immunomodulator (one intra-muscular injection)
IV	Acute mastitis (60 cows)	Intramammary antibiotics + nonsteroidal anti-inflammatory drug (one intramuscular injection)
V	Healthy (60 cows)	Control group: not treated

Milk samples were taken from cows with clinical mastitis without systemic signs and symptoms. Cows with acute mastitis were recognized by experienced veterinarians, who did not know the study design; the experiment was thus blinded. Clinical cases of udder inflammation manifested with hot and swollen mammary gland, abnormalities in milk (clots and watery discharge), decreased milk production, and positive California Mastitis Test (Mastirapid, Vetoquinol, France). Milk samples were collected before treatment and after cleaning the teats, discarding a few streams of milk and scrubbing the teat ends with cotton balls moistened with 70% alcohol. Medicines were injected on the first day of therapy by another group of veterinarians.

**Fig. 1:**
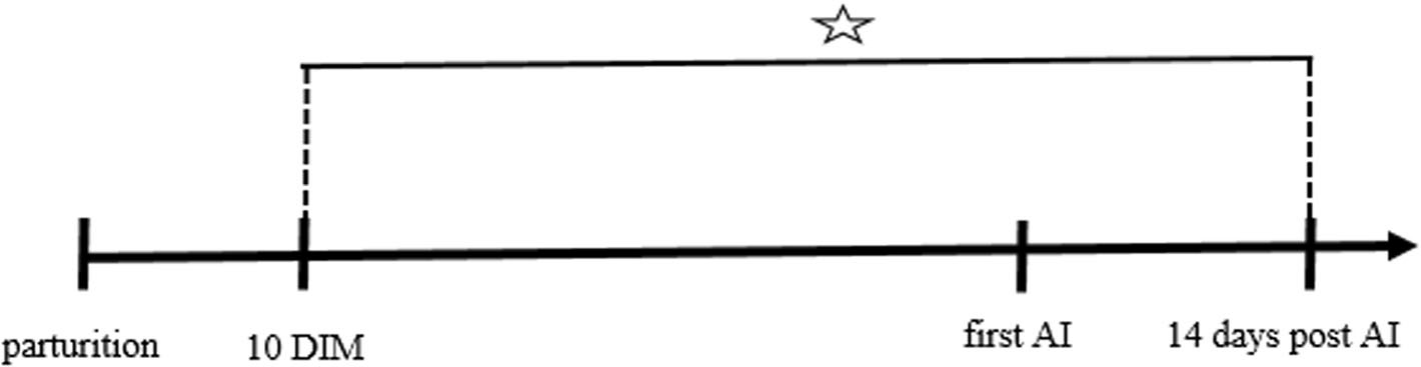
General experiment design. DIM: days in milk. AI: artificial insemination. ☆: period in which clinical mastitis was monitored for

Clinical mastitis was diagnosed from day 10 in milk to day 14 after the first AI. (Fig. 1). Each mastitic cow was systematically allocated to one of the four experimental groups (I–IV). In other words, the first mastitis case was allocated to the first treatment group, the second case to the second group, the third to the third, the fourth to the fourth, and the fifth mastitis case was then allocated to the first treatment group, and so on until each treatment group contained 60 cows. Sample size calculation was based on the study of Lents et al. [34]. Clinical cases of mastitis caused by yeasts or *Prototheca* spp. were excluded from the experiment. One control healthy cow to every four mastitic cows with no more than 14 days between calving were included on each farm. On day 1, 7, and 21 of diagnosed mastitis, quarter milk samples from affected cows were collected aseptically in order to perform microbiological culturing and antibiotic susceptibility assessment. Milk samples collected on day 21 of treatment were also analyzed for somatic cell counts (Fossomatic 90, Foss, Denmark). Bacteria were identified following the generally accepted procedure of agar plate milk microbiology test [35]. Antimicrobial susceptibility testing was performed using the disc diffusion method (Kirby-Bauer method) [36] on Mueller-Hinton agar according to Clinical and Laboratory Standards Institute guidelines. The antimicrobials discs investigated included *i.a.* 30 μg cefquinome, 2 μg clindamycin, 30 μg neomycin and 20/10 μg amoxicillin with clavulanic acid [35, 36].

The effectiveness of antibiotic therapy was assessed by observing the reduction in clinical symptoms (hot and swollen quarters) and the appearance of macroscopic milk changes (clots, watery secretion, serum purulent, or purulent secretion) during the first three to 7 days of therapy. The CM was considered cured when clinical signs had diminished by day 7 of therapy and a lack of clinical signs and bacteriological cure were observed on day 21. If more than one quarter was affected, the cow was considered cured only when all affected quarters lacked clinical and bacteriological observations. In some cases, antibiotic therapy was continued (with the second or next antibiotic treatment being described as prolonged treatment), which means the same antibiotic as used earlier in the particular treatment was applied three times every 12 hours on days 7 and/or 14 day of treatment. Cows were considered as not having recovered if, on day 21 after diagnosis, despite the multiple administrations of intramammary antibiotic, microorganisms could be cultured from milk samples, and if somatic cell counts in the milk were above 800,000/ml.

When udder inflammations were detected in the control group, the affected cows were excluded, each being replaced by another healthy cow with a similar calving date and without clinical mastitis and low somatic cell counts in milk (< 400,000/ml). In the experimental groups, additional therapy was provided alternately and randomly at the onset of antibiotic therapy. The additional therapy consisted of a single intramuscular injection of antioxidant Se and vitamin supplements at the dose recommended by the manufacturers (Table 8).

The investigation of reproduction performance continued until pregnancy was confirmed by *per rectum* examination, or the cows were culled. The following fertility indices were calculated for both control and experimental:

- conception rate after first AI (percentage of pregnant cows after first AI);
- days open (number of days from calving to next pregnancy);
- number of AIs per conception - i.e., the insemination index (number of AI procedures/pregnancy).

Owing to the fact that the cows were maintained in the routine production cycle after the study the animals were released and continued milk production.

### Biochemical analysis

On days 0 and 7 after the beginning of therapy, blood samples were collected from five cows in each group. Total antioxidant status was determined using a standard test kit (Randox Laboratories, UK, cat. no. NX 2332) and colorimetrically (Epoll 20 spectrophotometer, Poll, Poland). Calibration was made performed following the instructions provided by the manufacturer (Randox Quality Control).

### Statistical analysis

Selected parameters (conception rate after first AI, days open, number of AI per conception) were analyzed by one-way ANOVA using SPSS (IBM SPSS version 24.0). Normality was tested using Kolmogorov–Smirnov analysis, and outliers were removed from the database. The parameters were all normally distributed. Logistic regression was used to estimate odds of calving using maximum likelihood estimation. The ANOVA procedure was used to compare the control vs. treatment groups, control vs. cows with one treatment cycle or prolonged therapy, and control vs. all cows treated with antibiotic. The type of antibiotic was initially included in the model, it was not proved significantly, and therefore was finally excluded from the model. Significant differences between groups within the same parameters (*p* < 0.05) were tested using Tukey’s post-hoc test and are indicated using superscripts. Results are presented with the arithmetic average, standard deviation, percentage of samples/animals in groups, and level of significant differences.

## Data Availability

The datasets used and analyzed in the current study are available from the corresponding author on reasonable request.

## Abbreviations

NSAID: Nonsteroidal anti-inflammatory drug
ROS: Reactive oxygen species
CL: Corpus luteum
CM: Clinical mastitis
DIM: Days in milk
CR: Conception ratio
DO: Days open
NSC: Number of services per conception
AI: Artificial insemination
TAS: Total antioxidant status
SCC: Somatic cell counts
FT: First treatment
PRO: Prolonged treatment
Qr: Quarters
*S. aureus*: *Staphylococcus aureus*
CNS: Coagulase-negative staphylococci
G-: Gram-negative bacteria
*Str. sp*.: *Streptococcus* species
*S. agalactiae*: *Streptococcus agalactiae*
*S. uberis*: *Streptococcus uberis*
*E. coli*: *Escherichia coli*
PGF2α: Prostaglandin F_2α_
